# EVERYDAY MARKERS OF PERSONALITY IN ADULTHOOD

**DOI:** 10.1093/geroni/igz038.2858

**Published:** 2019-11-08

**Authors:** Stacey B Scott, Andreas Neubauer, Joshua Smyth, Martin Sliwinski

**Affiliations:** 1 Stony Brook University, Stony Brook, New York, United States; 2 DIPF, Frankfurt, Hessen, Germany; 3 Penn State, University Park, Pennsylvania, United States

## Abstract

Personality should manifest everyday as behaviors, affective states, and thoughts. Trait personality measures require retrospection and appraisal, processes affected by cognitive changes. Ecological momentary assessments (EMA), however, may identify sensitive everyday personality markers. We analyzed data from 178 individuals aged 20-79 who completed 3 EMA measurement bursts. Each burst, participants rated positive affect (PA), negative affect (NA), negative thoughts (URT), total social interactions, and interaction pleasantness up to 5x daily for 7 days. We tested for measurement invariance across bursts in a confirmatory factor analysis using 4 indicators of Neuroticism (N; a trait measure of N from BFI and 3 EMA-based indicators: mean NA, standard deviation NA, mean URT) and 4 indicators of Extraversion (E; trait E from BFI and mean PA, mean interactions, mean pleasantness). Strict measurement invariance held, indicating that the association among these indicators remained stable across the 18 month observation period.

